# The Features of Copper Metabolism in the Rat Liver during Development

**DOI:** 10.1371/journal.pone.0140797

**Published:** 2015-10-16

**Authors:** Yulia A. Zatulovskaia, Ekaterina Y. Ilyechova, Ludmila V. Puchkova

**Affiliations:** 1 Department of Biophysics, Institute of Physics, Nanotechnology, and Telecommunications, Peter the Great St. Petersburg Polytechnic University, St. Petersburg, Russia; 2 Department of Molecular Genetics, Institute of Experimental Medicine, St. Petersburg, Russia; 3 Laboratory of trace element metabolism, ITMO University, St. Petersburg, Russia; Karolinska Institutet, SWEDEN

## Abstract

Strong interest in copper homeostasis is due to the fact that copper is simultaneously a catalytic co-factor of the vital enzymes, a participant in signaling, and a toxic agent provoking oxidative stress. In mammals, during development copper metabolism is conformed to two types. In **e**mbryonic **t**ype **c**opper **m**etabolism (ETCM), newborns accumulate copper to high level in the liver because its excretion *via* bile is blocked; and serum copper concentration is low because ceruloplasmin (the main copper-containing protein of plasma) gene expression is repressed. In the late weaning, the ETCM switches to the **a**dult **t**ype **c**opper **m**etabolism (ATCM), which is manifested by the unlocking of copper excretion and the induction of ceruloplasmin gene activity. The considerable progress has been made in the understanding of the molecular basis of copper metabolic turnover in the ATCM, but many aspects of the copper homeostasis in the ETCM remain unclear. The aim of this study was to investigate the copper metabolism during transition from the ETCM (up to 12-days-old) to the ATCM in the rats. It was shown that in the liver, copper was accumulated in the nuclei during the first 5 days of life, and then it was re-located to the mitochondria. In parallel with the mitochondria, copper bulk bound with cytosolic metallothionein was increased. All compartments of the liver cells rapidly lost most of their copper on the 13^th^ day of life. In newborns, serum copper concentration was low, and its major fraction was associated with holo-Cp, however, a small portion of copper was bound to extracellular metallothionein and a substance that was slowly eluted during gel-filtration. In adults, serum copper concentration increased by about a factor of 3, while metallothionein-bound copper level decreased by a factor of 2. During development, the expression level of *Cp*, *Sod1*, *Cox4i1*, *Atp7b*, *Ctr1*, *Ctr2*, *Cox17*, and *Ccs* genes was significantly increased, and metallothionein was decreased. *Atp7a* gene’s activity was fully repressed. The copper routes in newborns are discussed.

## Introduction

Copper is an essential trace element belonging to transition metals. Due to its ability to change redox states Cu(I)↔Cu(II) and competence to form coordination spheres with amino acids that comprise–SH,–NH_2_,–COOH,–SCH_3_, and imidazole groups, copper is utilized by the enzymes controlling a wide range of cellular processes: oxidative phosphorylation, detoxification of reactive oxygen species (ROS), post-translation processing of collagen, elastin and neuropeptides, synthesis of neurotransmitters as well as bidirectional transfer of iron ions across plasma membranes [1, 2, 3]. But copper ions outside of the pre-organized coordination sphere can initiate Fenton type reactions and produce ROS [4]. The safe intracellular traffic of copper is provided by a specific protein system, which is highly conserved in evolution. Several families of proteins are emerging that help to confine copper to vital roles. They include integral membrane transporters, P-type Cu(I)/Cu(II)-ATPases, and soluble cytoplasmic Cu(I)-chaperone that package copper and guide it to apo-cuproenzymes. The members of this system contain high affinity Cu(I)-binding motifs with lower coordination numbers. They can easily pass Cu(I) to each other down the free energy gradient [5]. Even insignificant changes in the structure of the copper-transporting proteins, mutations in cuproenzymes as well as disturbance of copper homeostasis result in severe neurodegenerative diseases, cancer and other disorders [6–9]. Therefore, interest to molecular genetic mechanisms controlling the metabolism of copper is very high. The most valued studies are the *in vivo* investigations that use genetically modified animals with damaged genes of copper homeostasis [10–13]. As a rule such studies are carried out on animals in the early postnatal period of development and interpretation of the results do not always take into account that mammals have two natural types of copper metabolism. So in the liver of newborns, **e**mbryonic **t**ype of **c**opper **m**etabolism (ETCM) is retained. It is characterized by copper accumulation in the liver, which originates from two reasons: copper excretion through bile is blocked, and ceruloplasmin gene activity is very low. As a consequence, copper accumulates in the liver; and blood serum copper concentration stays low, as ceruloplasmin (Cp, a blue multicopper (ferro)oxidase and main extracellular copper-transporter, containing the ∼95% of serum copper) level is low. On the contrary, in the liver of adult mammals, copper excretion *via* bile is unblocked and the Cp gene activity is increased. As a result, liver copper concentration drops, and its serum level increases. The investigations of the ETCM features in comparison with the **a**dult **t**ype of **c**opper **m**etabolism (ATCM) were carried out in 1980’s–1990’s, when genes related to copper transport [14, 15] were not yet identified. So the present work revisited copper metabolism in the newborns taking into account the new data on genes controlling copper homeodynamics, which became available in the last years.

There is also another reason. For quite a long time the role of copper as a secondary messenger was not considered. Now, the fact that copper is a regulator of signaling, proliferation and apoptosis has been acknowledged [16–22]. To play the signaling role, copper needs to have an intracellular place, in which it can be retained, from which it can be recruited and quickly returned back. Convincing data on the pool of copper, which could provide its regulatory function, are unknown. It is possible that the ETCM is a suitable model, which will promote the understanding how the copper-dependent regulation works.

In this work, copper distribution in the compartments of hepatocytes, blood serum copper status indexes, the expression levels of genes coding for cuproenzymes, copper transporting proteins as well as copper-binding proteins were compared in the liver before and after the switching from ETCM to ATCM. The data may promote the understanding of the details of copper turnover that would help to correct the disorders associated with the disturbances of copper metabolism.

## Materials and Methods

### Animals and tissue samples

Two-month-old outbred adult albino rats purchased from Rappolovo (Leningrad Region, Russia) and rats born in the vivarium of the Institute of Experimental Medicine were used. No more than 10 animals or one female with litter were kept in plastic cages (1815 cm^2^ and 720 cm^2^, respectively) with wood shavings. Eight newborn rats were kept per female from a litter. The animals were housed with a 12:12-h light-dark cycle in 60% humidity and had free access to suitable food and water.

The biological samples were collected, snap frozen, and stored at –80°C for assessment of expression of genes and proteins as well as for metals concentration analysis. Blood samples were collected from the cervical vessels, clotted and then sera were separated by low-speed sedimentation.

### Ethics statement

Procedures involving animals and their care were conducted in conformity with institutional guidelines that are in compliance with national laws (Russian Federation the Ministry of Health N267, June 19, 2003; Guide for the Use of Laboratory Animals, Moscow, 2005). The studies were approved by the local Committee of Ethics at the Institute of Experimental Medicine (the Protocol number N2/13 approved 27^th^ of June 2013, Pavlov str., 12, St. Petersburg, 197376 Russia). The animals were sedated with diethyl ether vapor and euthanized by cervical dislocation, performed by skilled personnel. The animals, which were used for isolation of the liver mitochondria, were anesthetized with 1 g of oxybutyrate per 1 kg of body weight, since ether significantly disturbs the native state of mitochondrial membranes.

### Subcellular fractions

Subcellular fractions were isolated by differential centrifugation. The tissue samples were homogenized (1:6 w/v, respectively) in buffer A, containing 250 mM sucrose, 100 mM KCl, 5 mM MgCl_2_, 10 mM Tris-HCl (pH 7.4), 5 mM DTT, and 0.5 μl/ml of protease inhibitor cocktail (Sigma, USA), using T10 basic homogenizer for 3×20 s at maximum power (IKA, Germany). The homogenate was filtered through 6 layers of cheesecloth to discard the unbroken tissue fragments and centrifuged at 800×g for 10 min. The resultant pellet consisted of nuclei and plasma membrane fragments, which contained ~80% of total cellular ouabaine-sensitive Na/K-ATPase activity [23]. To estimate the distribution of copper between nuclei and closed plasma membrane fragments, the 800×g pellet was resuspended in buffer A (1:6 w/v), then loaded onto a 1.5 M sucrose cushion, and centrifuged at 15,000×g for 2 h. The pellet (nuclei) was collected, resuspended, and the copper concentration was determined as percentage of copper content in the 800×g fraction. More than 90% of copper were found in the nuclei. Subsequently 800×g pellet was used for determination of nuclear copper concentration. A crude mitochondrial fraction was isolated from the post-nuclear supernatant as sediment after centrifugation at 12,000×g for 20 min. To separate mitochondria, lysosomes and peroxisomes the pellet was resuspended, layered onto a stepwise density gradient 27–42–45–47–50% (w/w) of sucrose, and centrifuged at 58,000×g for 5 h (SW27 rotor, Optima LE-80K centrifuge, Beckman, USA). To identify organelles 1-ml fractions of the gradients were collected and the sucrose density was measured using Refractometer-IPF-454B2M, Russia. The fraction collected at the border of 45% sucrose (density of 1.18–1.19 g/cm^3^) was positive for cytochrome-c-oxidase subunit 4 isoform 1 (Cox4il) in Western blot (WB) assay and MitoTracker Red 580 staining [24]. This fraction was considered mitochondria. The material collected at the border of 47% sucrose (density of 1.20 g/cm^3^) was shown to be enriched with acid phosphatase. Enzymatic activity of acid phosphatase was determined as previously described [25]. Briefly, to detect acid phosphatase the fraction was incubated with *p*-nitrophenyl phosphate in 50 mM citrate buffer, pH 4.8, for 30 min at 25°C. Reaction was terminated with 50 mM NaOH; and absorbance at 405 nm was measured. The abundance of acid phosphatase activity was associated with ∼1.20 g/cm^3^ fraction, which was then considered to comprise lysosomes. The fraction at the border of 50% sucrose with a density of ∼1.22 g/cm^3^ was collected and considered to be peroxisomes without additional testing. Fractions with mitochondria, lysosomes and peroxisomes were diluted 4-fold and the pellets were collected by centrifugation at 15,000×g for 30 min. A total intracellular membrane fraction (endoplasmic reticulum + Golgi complex) was isolated from the post-mitochondrial supernatant as sediment at 23,000×g, 60 min. The supernatant of the last centrifugation comprised the cytosolic fraction. In samples, protein and copper concentrations were measured and the results were expressed as μg Cu/mg protein.

### Measurement of relative level of mRNAs

Total RNA was isolated using TRIzol Reagent (Invitrogen, UK). RNA concentration was measured using a NanoDrop 2000 spectrophotometer (Thermo Scientific, USA) following the standard procedure. The purity of RNA samples was proved by the optical density ratio A_260_/A_280_>1.8. To verify the integrity of the samples, the 18S/28S RNA ratio was analyzed after electrophoresis in 1.4% agarose gel. Design of primers was performed using the Primer-BLAST software (NCBI, USA); the primer sequences, sizes of PCR products and annealing temperatures are presented in the Table 1. For each pair of primers the concentrations of primers and MgCl_2_, annealing temperature, and time setup as well as appropriate number of cycles for semi-quantitative PCR were optimized using MJ Mini Personal Thermal Cycler (BioRad, USA). As a result, 25 pM of each primer and 3 mM MgCl_2_ were used for all amplifications. β-actin was selected as the internal control because its mRNA levels during development did not vary significantly. PCR consisted of the following steps: initial denaturation (5 min at 94°C), cycles of amplification (denaturation – 1 min at 94°C, annealing of primers – 1 min, elongation – 1 min at 72°C) and terminal elongation (7 min at 72°C). Amplification included 28 cycles for β-actin and 30 cycles for all other genes. The electrophoretic analysis of the PCR products demonstrated that their sizes corresponded to the calculated values and non-specific products were not synthesized under the chosen experimental conditions. RT-PCR products were analyzed in a 1.4% agarose gel with ethidium bromide and the data processed using ImageJ software. The results were expressed in arbitrary units (a. u.) as a ratio between the amount of the PCR product of the mRNA specified and the amount of the PCR product of β-actin obtained with the same RNA preparations under similar conditions. The results are given as bar charts. Each value was combined from 3 independent PCR replicas of cDNA samples, obtained from 4 animals.

**Table 1:** Sequences of primers used for RT-PCR analysis.

Gene*	Nucleotide sequence (5'→3') of primers	Product size, bp	T
***Atp7a***	F: gaa gcc tac ttt ccc ggc tac aac aga agc	421	64
	R: agg tac cca agg ttt cag tgt cca gct cc		
***Atp7b***	F: cag aag tac ttt cct agc cct agc cct agc aag c	332	65
	R: ccc acc aca gcc aga acc ttc ctg ag		
***β-actin***	F: gaa gat cct gac cga gcg tg	327	59
	R: agc act gtg ttg gca tag ag		
***Ccs***	F: cag tct ggt tgt tga tga ggg aga ag	265	60
	R: act gaa taa cct gac agg agg ctc tg		
***Commd1***	F: gag ggg aat tct caa gtc tat tgc	317	60
	R: ctc aga ttc ccg tcc act tct c		
***Cox4i1***	F:aag aga gcc att tct act tcg gtg tg	484	60
	R: cag gct ctc act tct tcc att cat tc		
***Cox17***	F: ctc ggg ttg gtc tga gtt ttg	308	59
	R: tac tct tct tca ttc ttc agg gct t		
***Cp***	F:agt aaa caa agt cac aac gag gaa t	398	57
	R:tcg tat tcc act tat cac caa ttt a		
***GPI-Cp***	F:agt aaa caa agt cac aac gag gaa t	436	57
	R: ctc ctt ggt aga tat ttg gaa taa a		
***Mt1a***	F: cga ctg cct tct tgt cgc tta cac c	350	58
	R: tca cat gct cgg tag aaa acg ggg t		
***Slc31a1 (Ctr1)***	F: tgc cta tga cct tct act ttg g	358	57
	R: atg aag atg agc atg agg aag		
***Slc31a2 (Ctr2)***	F: gag gct gtg ctt ctc ttt gat t	203	60
	R: gag cct gta gaa tcc tgg tct g		
***Sod1***	F:aca ata cac aag gct gta cca ctg cag g	220	62
	R: tca tct tgt ttc tcg tgg acc acc ata g		

*The gene names are given according to rat genome databases in alphabetical order.

F–forward, R–reverse. T-annealing temperature, °C.

### Immunoblotting

For WB analysis the samples were equalized for protein content; and the electrophoresis of proteins was performed in either 8% or 15% polyacrylamide gel (PAG) with 0.1% SDS following Laemmli method. Proteins were transferred onto a Hybond ECL nitrocellulose membrane Amersham™ Hybond™- ECL ‘‘GE Healthcare” (USA). The quality and uniformity of protein transfer were controlled by total protein staining with Ponceau S, blocked with 5% non-fat milk, and blotted with the primary antibodies. The bands were visualized by enhanced chemiluminescence using ECL reagent and ECL Hyperfilm Amersham™ Hyperfilmt™ ECL ‘‘GE Healthcare” (USA). Densitometric quantitation of WB analysis was performed using MochaTM (Jandel Science) or Image Studio Lite (LI-COR Biosciences). For densitometry, a horizontal band running through the center of the gel (approximately corresponding to 50 kDa) was selected and used further for normalization [26]. Molecular mass markers from 14.4 to 116 kDa “ThermoScientific”, cat. N26610 (USA) were used to determine protein molecular weight.

### Immunoprecipitation

Cp was precipitated from 50 μl of rat serum (∼10–35 μg Cp) by 500 μl (1 mg/ml) of IgG isolated from serum of the rabbits immunized with rat Cp. The used ratio of Cp and antibodies corresponded to the specific and complete precipitation according to immunoelectrophoresis [27]. Precipitation was carried out overnight at 4°C, with continuous rocking. Then mixtures were centrifuged at 10,000×g for 10 min; the pellets were washed twice with PBS, collected by centrifugation, and then dissolved in 400 μl of pure nitric acid.

Precipitation of serum metallothionein (MT) included two steps. First, 5 μl of antibodies to MT were added to 300 μl of rat serum and the mixture was incubated overnight at 4°C with constant shaking. Then 10 μl of rabbit serum and 170 μl of donkey anti-rabbit IgG (secondary antibodies, 1 mg/ml) were added to the mixture. Rabbit serum (10 μl) was used as an IgG source to enhance the second stage of precipitation. After an overnight incubation, mixtures were treated as described previously.

### Antibodies

Polyclonal rabbit antibodies specific to rat Cp were used for Cp detection [27]. Rabbit antibodies to SOD1 and COX4i1 were obtained from Abcam (UK). Antibodies to MT (Santa Cruz Biotechnology, USA) were rabbit polyclonal IgG (200 μg/ml) against the epitope corresponding to residues 1–61 of the full length human MT-1H recommended for detection of all MT isoforms in a broad range of mammalian species. Goat antibodies to COMMD1 were purchased from Santa Cruz Biotechnology (USA). Goat anti-rabbit (Abcam, UK) and donkey anti-goat (Santa Cruz Biotechnology, USA) IgG, conjugated with horseradish peroxidase, were used as secondary antibodies. The immune complexes were visualized by ECL kit (Amersham™ Hybond™- ECL “GE Healthcare”, USA).

### Rocket immunoelectrophoresis

Cp protein concentration was measured by rocket immunoelectrophoresis exactly as described previously [27]. Briefly, a 1% agarose gel, containing 100 μg/ml antibodies to Cp, was prepared in electrode buffer (187 mM Tris, 374 mM glycine, 5.6 mM barbital, 32 mM barbital sodium, pH 8.8). 3 μl serum aliquots were loaded into wells. Each aliquot was a mixture of equal volumes of the sera from three rats. Electrophoresis was carried out overnight (10 V/cm) at 10–15°C. The gels were pressed with several layers of filter paper, and then dried completely with a heat dryer. Immune zones were visualized by *o*-dianisidine staining. The area of the precipitation peak was measured as the area of an isosceles triangle. Standard solutions of different Cp concentrations were run on each gel. Rat Cp (A_610_/A_280_ = 0.045) was used as a quantitative standard. The quantity of Cp in the samples was determined according to the standard curves.

### Gel-filtration chromatography

Blood serum samples (2 ml) or cytosolic fractions (2 ml containing about 40 mg total protein) were fractionated on the column with Sephadex G-75 in phosphate saline buffer, pH 7.4 or buffer A, respectively. The void volume of the column was estimated using blue dextran. Horse cytochrome *C* was used as a molecular weight marker (Sigma, USA). The fractions following the void volume (~1.5 ml per fraction) were collected and specified by D_280_ and D_254_.

### Biochemical assays

Specific activities of cuproenzymes were detected by assay-in-gel methods. After non-denaturating PAGE, the gels were stained with: *o*-dianisidine to determine the oxidase activity of Cp [28]; Mohr salt/ferrozine system for Cp ferroxidase activity [29]; or nitro blue tetrazolium for SOD activity [30]. Protein concentration was determined by Bradford assay using BSA as a standard. To reveal total protein bands gels were stained with Coomassie G250 or AgNO_3_ according to standard methods.

### Metal concentration measurement

Atomic copper concentration was measured by FAAS with electrothermal atomization and Zeeman correction of non-selective absorption by ZEEnit 650P spectrometer (AnalytikJena, Germany). The samples were dissolved in pure HNO_3_ or in a mixture of NaOH and SDS as described earlier [27]. In blood serum, copper concentration was measured without additional pretreatment. All solutions were prepared in deionized water pretreated with Chelex-100 resin.

### Statistical analysis

Data are expressed as means ± SD. Inference about changes was carried out by unpaired two-tailed Student’s *t*-test; the changes were considered significant at *p* <0.05.

## Results

### Copper concentration and its distribution in the liver during postnatal development

**Fig 1:**
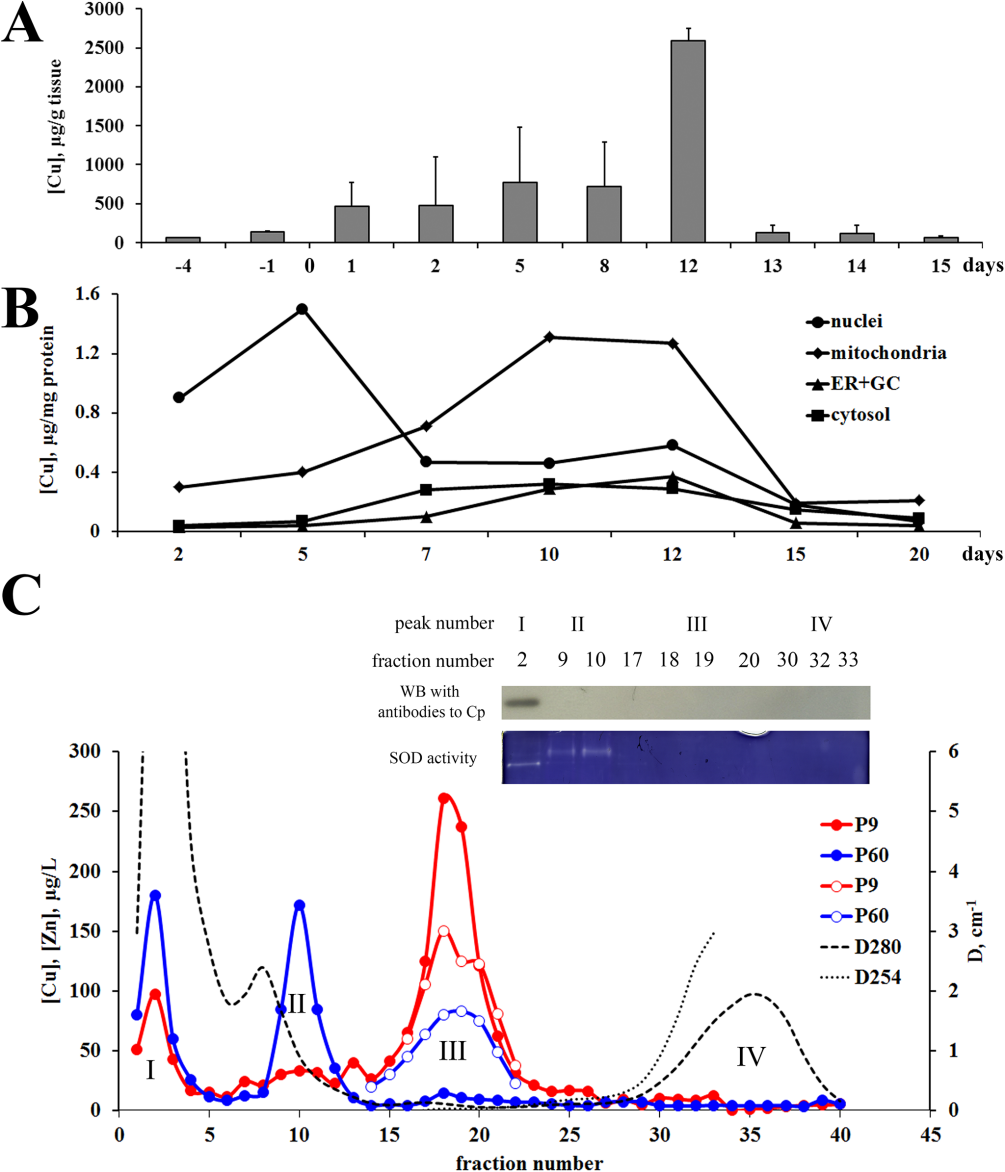
Hepatic copper concentration and copper distribution in the liver cells of newborn rats. (А) *Copper is accumulated in the liver from embryonic stage development to 12* ^*th*^ *day of life*. Ordinate axis: hepatic copper concentrations, μg/g wet weight (the means ± SD, *n =* 5); abscissa axis: age, days. (B) *During accumulation copper is redistributed between subcellular compartments*. Each dot represents the average from 3 experiments, in which the differences were not more than 10%. For each point, depending on the age of rats, 2–10 livers were combined to isolate subcellular fractions (nuclei–circle, mitochondria–rhomb, (endoplasmic reticulum + Golgi complex)–triangle, cytosol–rectangle). Ordinate axis: copper concentrations, μg/mg total protein; abscissa axis: age, days. (C) *Gel-filtration distribution of copper in cytosol of the newborn (P9*, *red) and adult (P60*, *blue) rats*. Cytosolic fraction was isolated from about 1 g liver tissue (a mixture of ∼350 mg of liver tissue from three rats) as described in Methods. Ordinate axis *(left)*: copper (closed dots) or zinc (open dots) concentration, μg/L, Arrow shows eluted position of cytochrome *C*. Ordinate axis *(right)*: optical density–D_280_ (dotted line) and D_254_ (dots). Abscissa axis: fraction number. Inset: WB with antibodies to ceruloplasmin (8% SDS-PAGE) and SOD activity (gel-test, 8% PAGE); the samples content 10 μl of the major copper fractions from peaks I, II, III, and IV of P9 cytosol.

To test the ontogenetic-required changes of copper metabolism, copper concentration was determined in the rat liver tissue and subcellular organelles during the first 15 days of life and compared with adults. Results are summarized in Fig 1. They demonstrate that a progressive copper accumulation began at the embryonic stage of development (Fig 1A) and lasted until the 12^th^ day of postnatal life (P12). Then copper concentration sharply dropped to the level that is normal for the adult rats. These results are entirely consistent with earlier data [14, 15], which formed the basis for a common concept that the rats to 12^th^ day of life retain the ETCM, which is characterized by the accumulation of copper in liver. After the 13^th^ day of life, the ETCM is switched to the ATCM, and copper is not accumulated in the liver anymore but it is excreted through the bile or included to Cp that was secreted to bloodstream. However an intracellular place copper accumulation is not defined precisely in earlier works. To obtain such data copper concentration in subcellular fractions from 1^st^ to 20^th^ days was measured (Fig 1B). It was shown that during the ETCM copper was re-distributed between the compartments of the hepatic cells. So, after birth copper was accumulated in the nuclei, but after 5^th^ day of life copper concentration in the nuclei decreased. Simultaneously, the specific copper content increased in mitochondria, thereby reaching the peak value in P12 rats. As total mitochondrial fraction isolated by differential centrifugation comprises lysosomes and peroxisomes it did not confidently indicate the exact site of copper accumulation. So the mitochondrial fraction was additionally fractionated by equilibrium ultracentrifugation in a stepwise density gradient of sucrose. Isolated fractions were identified as described in Methods and recognized as mitochondria, lysosomes and peroxisomes, and copper concentration was separately measured in them. It was determined that the purified fraction of mitochondria contained about 90% of copper from the crude mitochondrial fraction. So, our study provides a strong evidence to consider mitochondria as a copper deposit organelle during ETCM. Furthermore, copper concentration increased in cytosolic and intracellular membrane fractions in P5 to P12 rats (Fig 1B). The shape of curves for the changes of copper content in these fractions practically coincides with the mitochondrial curve, but copper content in these organelles was strongly lower. After the P12, copper concentration decreased in all cell compartments (Fig 1B).

**Fig 2:**
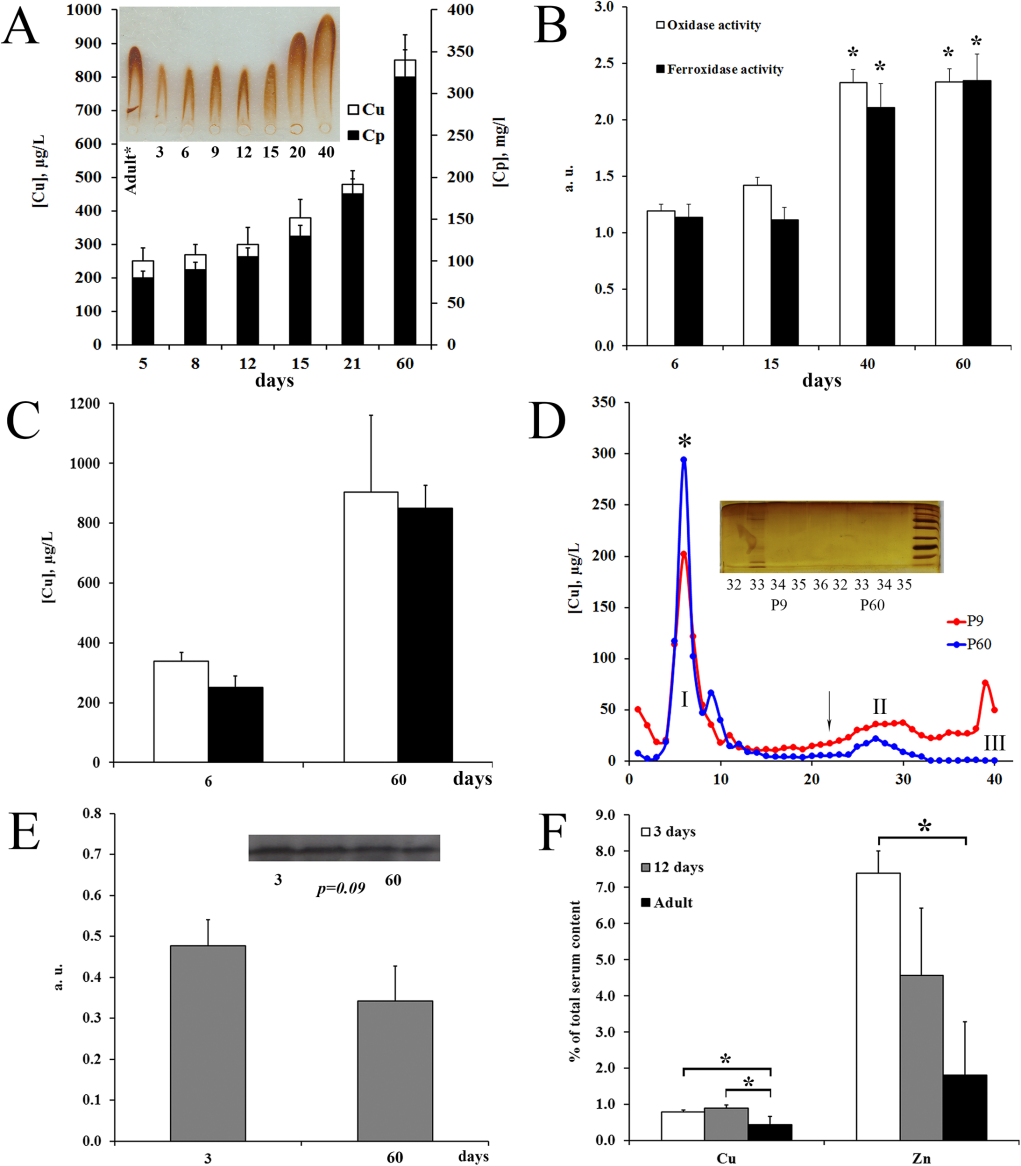
Ontogenetic changes of copper balance in the blood serum of rats. (A) *Serum copper concentration and ceruloplasmin protein level increased coordinately during development*. Ordinate axis: (left) copper concentration, μg/L (light bars), (right) ceruloplasmin protein concentration measured by rocket immunoelectrophoresis, mg/L (black bars), the means ± SD, *n =* 5. Inset: the representative protocol of immunoelectrophoresis. *Serum of adult rat was 2-fold diluted. Abscissa axis: age, days. (B) *Blood serum oxidase and ferroxidase activities increased after ETCM→ATCM transition*. Enzymatic activities were determined by gel-assay and expressed as a. u., the means ± SD, *n =* 3. Abscissa axis: age, days, *Р<0.05 in comparison with newborns. (C) *In the serum of newborn and adult rats*, *the main copper portion was precipitated with ceruloplasmin*. Ordinate axis: copper concentration, μg/L, light bars–actual serum copper concentration; black bars–copper concentration in ceruloplasmin precipitates. (D) *Gel-filtration distribution of blood serum copper of P9 (red) and P60 (blue) rats*. Ordinate axis: cooper concentration, μg/L; abscissa axis: gel-filtration fraction number. *—position of the maximum oxidase activity. Arrow show eluted position of cytochrome *C*. Inset: 12% SDS-PAGE of fractions from peak III of P9 and P60 rats. The samples were treated with SDS and 2-mercaptoethanol at 95°C, 5 min. Gel was stained with AgNO_3_. Right lane: molecular weight calibration markers from 3.4–100 kDa (ThermoScientific, cat. N26632, USA). (E) *Metallothionein (MT) presents in the serum of newborn and adult rats*. Upper: WB protocol of blood serum P9 and P60 rats with antibodies to MT (samples content 1.0 μl serum); below: relative level of MT in newborn and adult rats, the means ± SD *(n = 3)*. (F) *Serum* c*opper and zinc are precipitated by antibodies to MT*. Ordinate axis: Copper or zinc content in MT precipitates, % from atomic concentration copper or zinc in serum (*n* = 2).

In P9 and P60 rats, the distribution of copper between cytosolic proteins was compared by gel-filtration. In the both samples copper was distributed as three major peaks (Fig 1C). The first copper peak is associated with high molecular weight protein fraction. In this fraction according to WB analysis serum protein Cp is present (Fig 1C, inset). Perhaps, it was blood Cp, which contaminated the cytosol during homogenization. In newborns, the copper content of this peak is less than in adults. This is consistent with the serum low Cp level at the ETCM (see Fig 2A). In the same fraction (N2 fraction) SOD activity was determined (Fig 1C, inset). The SOD-band had different mobility than SOD-bands from fractions N9-10. Perhaps, SOD3 was presented in this peak, because its native molecular weight (∼130 kDa) corresponded to position of peak I. In peak II, copper concentration was higher in adults. SOD activity was associated with it (Fig 1C, inset). The position of this peak in the chromatogram with regard to cytochrome *C* and Cp, as well as the presence of SOD-activity support that the copper of peak II is associated with Cu(II)/Zn(II)-SOD1. In newborns, the copper concentration in the peak III is more that in the adults. The position of this peak (approximate molecular weight 15–8 kDa) as well as co-localization of zinc with its allowed us identify peak III as MT’s. In P9 rats, but not in adults, minor copper peak IV (fractions N30-35) was present. In these fractions copper concentration was above background level approximately by a factor of 2. Electrophoresis in 15% PAG under denaturing conditions did not reveal any material to be stained with silver nitrate or Coomassie G250.

### Changes of serum copper status during development

To evaluate the changes of copper status that depend on ETCM→ATCM transition the following serum indexes were measured: atomic copper concentration, Cp protein concentration, serum oxidase and ferroxidase activities, relative content of the MT protein, as well as the amount of copper bound with Cp and MT. The obtained data are illustrated by Fig 2. They show that, in newborns, the Cp protein concentration calculated from rocket immunoelectrophoresis (Fig 2A, inset) is about three times lower as compared to adults. Cp concentration did not increase immediately after P13, when the liver copper level dropped (see Fig 1A). Regardless of age, approximately 5–6 copper atoms corresponded to one Cp molecule. Both Cp enzymatic activities increased after ETCM→ATCM transition (Fig 2B). Immunoprecipitation data indicated undoubtedly that the major fraction of serum copper was associated with Cp in both newborn and adult rats (Fig 2C).

The comparison of copper distribution in the sera of P9 and P60 rats by gel filtration assay revealed that at both ages the major part of copper was localized in the high-molecular-weight fraction (Fig 2D). It coincided with the Cp peak, as confirmed by the oxidase activity test. The copper profile showed that the peak II position corresponding to MT was more in newborns (Fig 2D). It is known that MT is present in serum of adult mammals [31]. Outside the cell, it acts as a transporter of Zn and Cu, and participates in the support of their homeostasis. WB analysis demonstrated that MT was also present in the serum of newborns and its concentration was higher than in adult rats (Fig 2E). It appeared that about 1% of total serum copper was bound to MT and this quantity decreased by a factor of 2 after ETCM→ATCM switching (Fig 2F). In newborns, about 8% of the total serum zinc amount was also bound to MT, and the quantity of Zn decreased in adult animals (Fig 2F). At the same time the total zinc concentration decreased from ∼3500±230 to ∼1500±90 μg/L (*P*<0.05). The ontogenetic-dependent decrease of serum zinc concentration in rats is described for the first time; and this agrees well with the fact that zinc concentration in the human serum is reduced by half to the 8^th^ week of life [32]. The copper of peak III had no analog in adult rat serum. It is located in the region containing the low-molecular substances absorbing in D_254_ range. It contained substances that are heterogeneous by molecular weight and did not stain with Coomassie G250, but stained with silver nitrate (Fig 2D, inset). The major part of this substance was revealed in fraction N33, but its traces were detected in fractions N34 and N35.

### Comparison of the activity of genes encoding cuproenzymes and proteins associated with copper transport during development

In the work, three groups of genes were analyzed. *Group 1* included genes encoding cuproenzymes: Cp, the major blood multicopper blue (ferr)oxidase synthesizing in liver [33]; a splice isoform of Cp, GPI-Cp that has glycosylphosphatidylinositol anchor [34]; Cu(II)/Zn(II)-superoxide dismutase 1 (SOD1), the main cytosolic cuproenzyme [35]; and Cox4i1, the nuclear subunit of COX that is absolutely required for the assembly of physiologically mature complex IV of the electron transport chain [36]. *Group 2* comprised the genes encoding Cu(I)-transporters and carriers: CTR1, high affinity copper importer localized on plasma membrane [37]; CTR2, low affinity copper transporter integrated to the membranes of endosome/lysosome system [37]; CCS, a cytosolic Cu(I)-chaperone incorporating copper into apo-SOD1 [38]; COX17, cytosolic Cu(I)-chaperone involved in the recruitment of copper to mitochondria [39], and two Cu(I)/Cu(II)-transporting P-type ATPases (ATP7A and ATP7B) that provide copper excretion and its active transport from the cytosol to the Golgi complex lumen, where extracellular cuproenzymes are metallated [40]. *Group 3* contained MT isoform 1a (MT1a) gene for copper storage and its redox cycle system [41, 42]. Also the relative concentration of mature products of transcription of COMMD1 gene was measured [43]. This multifunctional protein involved in the copper excretion and regulation of various cellular activities [13, 44, 45].

**Fig 3:**
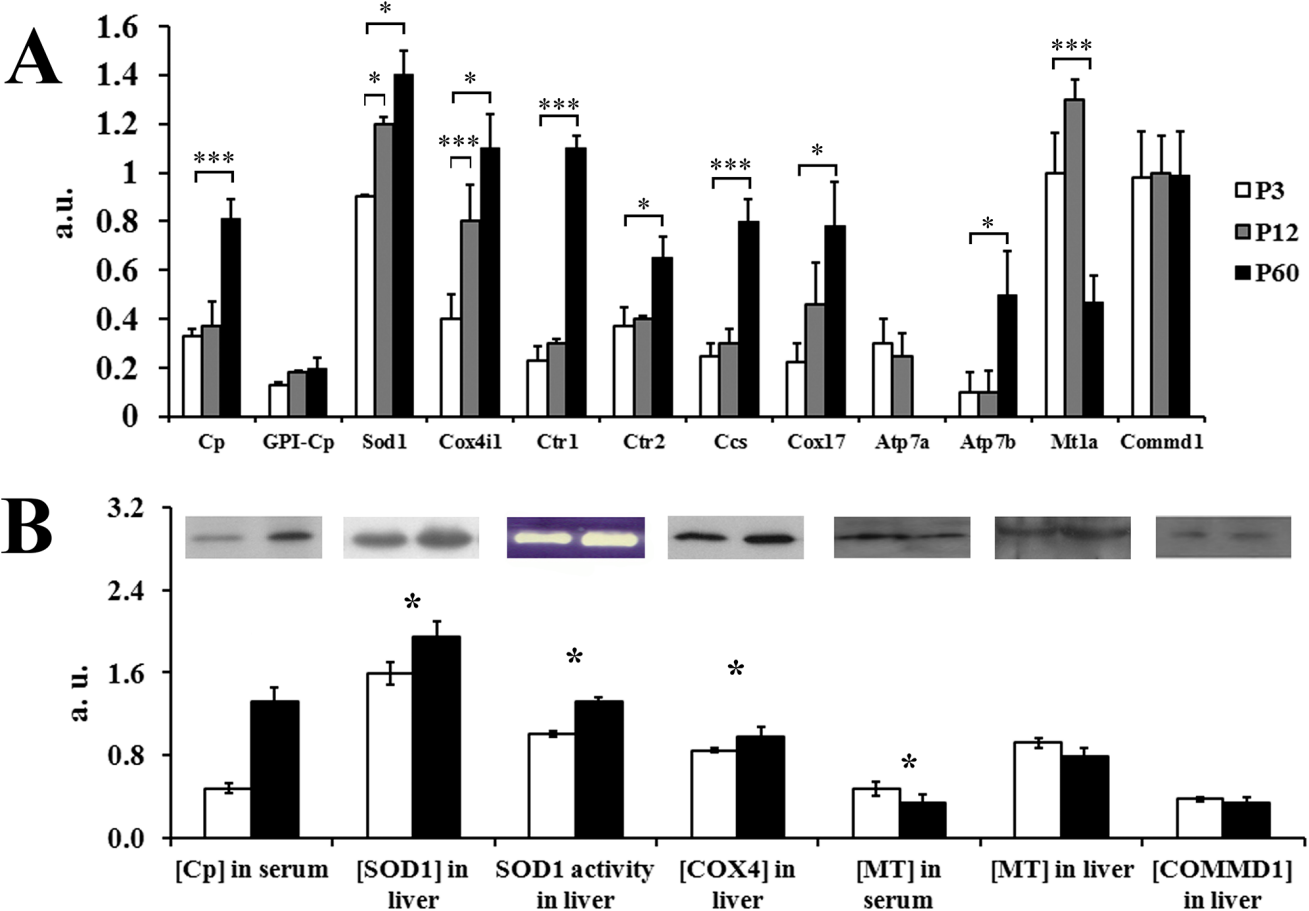
Hepatic expression of the genes associated with copper metabolism during ETCM→ATCM transition. (A) *RT-PCR analysis of the relative levels of mRNAs*. Ordinate axis: the data expressed as a. u., the means ± SD *(n = 4);* *—*P*<0.05, **—*P*<0.01, ***—*P*<0.005. Light bars–P3, grey bars–P12, black bars–P60. (B) *Western blot analysis of the relative content of proteins associated with copper metabolism*. Upper: examples of WB and SOD activity protocols. The molecular weight of WB identified proteins corresponds to: Cp ∼130 kDa (8% SDS-PAGE), SOD1 ∼17 kDa (12% SDS-PAGE), COX4 ∼20 kDa (12% SDS-PAGE), MT ∼8 kDa (15% SDS-PAGE), COMMD1 ∼23 kDa (12% SDS-PAGE); SOD activity was identified by gel-assay (blue gel) as described in Methods. Below: densitometric quantification of WB and SOD1 activity data. Abscissa axis: relative protein content, a. u., the means ± SD *(n = 4)*. Light bars–P9, black bars–P60 rats.

We assessed the activity of listed above genes in the liver of the P3 (copper accumulates in nuclei), P12 (copper accumulates in mitochondria), and P60 (adult) rats. Processed results of the semi quantity RT-PCR analysis are presented Fig 3A. They show that *Cp* gene activity increased after ETCM→ATCM transition. There was a positive linear correlation between Cp-mRNA level, copper concentration and serum Cp protein contents as well as with its enzymatic activities (Figs 3A, 3B, 2A and 2B). In adult rats, the splice-isoform encoding GPI-Cp appeared. It is known that GPI-Cp mRNA did not form in the HepG2 liver cell culture [46] and, perhaps, GPI-Cp was produced by non-hepatocyte cells (*e*.*g*., Kupffer cells), whose amount increased during development. Adult rats had significantly higher COX4i1-mRNA level and COX4i1 protein concentration than the newborns (Fig 3A and 3B). The relative level of SOD1-mRNA, its protein concentration, and enzymatic activity significantly increased after ETCM→ATCM switching (Fig 3A and 3B). Also Cox4i1 and Sod1 gene activities were significantly increased between the 3^rd^ and 12^th^ days of life.

At the same time in newborns, the gene expression level of *Atp7b*, *Cox17*, and *Ccs*, which metallated the mentioned cuproenzymes, was very low (Fig 3A). However *Ctr2* gene activity was higher than the *Ctr1* gene activity (Fig 3A). In the adult rats, the activity of these genes significantly increased. The relative concentration of MT1a-mRNA was up to 2-fold higher at the ETCM; and this is in good agreement with higher copper concentration in the cytosol of newborns (Fig 1C). However MT protein level decreased insignificantly after the ETCM→ATCM transition (Fig 3B). No ontogenesis related differences in the expression of Commd1 gene were found (Fig 3A and 3B).

## Discussion

The purpose of this work was to reduce the number of unknown aspects of the copper metabolism switch in rats during development. We will discuss the obtained data in the following theses.

### (i) In the liver, copper redistributes during ETCM

Earlier it was noted that normal mammalian newborns have high hepatic copper concentration and the physiologic deficiency of serum Cp [47–49]. The patients with Wilson disease demonstrate the same copper status phenotype [50]. Such copper status develops as a result of the low activity of the *Atp7b* gene (during the ETCM) or *Atp7b* mutations (Wilson disease) [40, 51]. So the ETCM may be viewed as a phenocopy of Wilson disease [52]. We have confirmed that in the liver, at the ETCM, copper was accumulated in large amounts (Fig 1A); and additionally showed that at the same time copper was redistributed between nuclei and mitochondria in age-dependent manner (Fig 1B). Interestingly that in the same ways copper is distributed in the liver of LEC line rats (having mutations in *Atp7b*) and genetically engineered *Atp7b* ^*-/-*^ knockout mice [12, 52–54]. In newborns, some part of copper is also accumulated by MT (Fig 1C). Meanwhile, Zn/Cu ratio in MT fraction of newborns was low. In adult rats the concentration of both metals in MT fraction decreased, but Zn/Cu ratio became higher.

In the mitochondria, copper is a structural and catalytic co-factor of COX. Copper for COX is transported by CTR1→COX17→SCO1/2(COX11)→COX1/COX2 transfer system [5]. Also a small amount of Cu/Zn-SOD1 is metallated in intermembrane space of mitochondria [55]. However, mitochondrial atomic copper concentration is an order of magnitude higher than the amount necessary for normal COX function and mitochondrial SOD1 [56]. It is mean that mitochondria have an alternative machine, which could provide influx/efflux of copper. Little is known about this mitochondrial copper way. Perhaps, copper is imported to the mitochondria by PiC2, the protein of the mitochondrial carrier family [57]. Possibly, copper is excreted by the mitochondrial isoform of ATP7B, which is produced as a result of the specific post-translational cleavage [58]. The role of a copper soluble carrier for both PiC2 and mitochondrial ATP7B may be played by a low-molecular-weight Cu(I)-carrier, which moves as a shuttle between the mitochondrial matrix and cytosol [56, 59]. This carrier was hypothesized to be similar to methanobactin, highly modified peptide of methanotrophs, which carries copper from the environment to bacteria [60]. Interesting that methanobactin decreased mitochondrial copper concentration in a rat model for Wilson disease [61]. It can be assumed that the mitochondria may act as a copper storage organelle in the liver at the end of ETCM, and possible they are a reservoir of copper, which is used in signaling.

At the same stage of ETCM, copper was bound by cytosolic MT (Fig 1C). MTs is form a family of ubiquitous cysteine-rich, low molecular weight (MW ranging from 5 to 14 kDa) proteins with pleiotropic functions and high affinity for trace elements like zinc and copper. In mammals, there are about 10 MT genes and even more protein forms are identified, but functions of the most of them remain poorly understood [62]. At present, evidence is accumulating that molecular forms of MT may have different function [63]. In the present work, MT1a isoform was selected for investigation because it is associated with copper homeostasis in the liver. In newborns, MT1a gene expression was significantly higher than in adults (Fig 3A). However, the protein level of MT was similar in both ETCM and ATCM (Fig 3B). It is possible that discrepancy between the levels of mRNA and protein appeared because of MT-mRNA level was measured using specific primers for MT1a isoform, while immunoprecipitation and WB were performed with commercial antibodies to MT, which cross-reacted with all forms of MT.

### (ii) In the blood of newborns, copper presents in extracellular MT and low molecular complex

The ETCM serum copper status indexes measured in this work (copper concentration, level Cp protein, oxidase and ferroxidase activities) were in good agreement with the earlier data, which demonstrated holo-Cp level was low in newborns [14, 15]. But still, three new facts came into notice. First, the top copper excretion from liver (P13) began substantially ahead of the increased synthesis and secretion of the Cp, which typically occurs after 20^th^ day of age (Figs 1A and 2A). This indicates that these processes, which have yet to be understood, are independent.

Second, MT is present in the serum of the newborns. More than half a century MT has been considered as an intracellular protein entirely, but in the last decade the data that MT presents in the extracellular spaces were published. MT was found in serum in increasing concentrations following restraint stress, and during exposure to toxic metals, as well as upon severe liver diseases and various tumors [64–66]. It was suggested that the MT was secreted by the Golgi-independent pathway [67]. In this work, WB analysis and immunoprecipitation showed exactly that MT circulated in bloodstream of newborns and it contained copper and zinc (Fig 2E and 2F). Since apo-MT would be rapidly oxidized in the extracellular environment, it could be loaded with trace elements inside the cell only. The data show that liver MT-mRNA level and MT protein concentration in the serum changed concertedly; and age-mediated changes of extracellular Cu-MT level correlate with contain of Cu-MT in the liver cytosol (Figs 2F and 3). It can be assumed that, in newborns, blood MT is the carrier of the copper from the liver to other organs. However the organ of origin of blood MT as well as its physiological role remains unknown.

Third, in newborn serum, copper-containing complex, which eluted very slowly and dissociated after treatment with denaturing agents, was found (Fig 2D, inset). Since the complex absorbs at D_254_ and its components are stained with AgNO_3_, but not Coomassie, possible that it includes ribokines, copper-regulated extracellular RNA chaperone-shaped protein assemblies [68, 69]. Earlier a small copper carrier has been found in the serum and urine of patients during the late stages of Wilson disease [70]. Chemical nature of the complexes have not determined yet.

### (iii) At the ETCM, the expression profile of cuproenzymes does not correlate with expression level of Cu-chaperons that metalized their

Expression of the major essential cuproenzymes of vertebrates (extracellular Cp, cytosolic SOD1 and mitochondrial COX) has been studied at the transcriptional and translational levels, as well as at the level of enzymatic activity for switching from ETCM to ATCM (Fig 3). As shown by the RT-PCR analysis, WB assay, and enzymatic tests for these enzymes, the levels of mRNAs, their translation products and enzymatic activities are fully consistent (Fig 3A and 3B). The cuproenzymes were expressed in early ETCM and their expression significantly increased after the ETCM→ATCM transition. Also we observed the *Atp7a* gene repression and *Atp7b* gene activation at the end of ETCM (Fig 3). This observation agrees with the data obtained earlier in other laboratories [71, 72].

In this work, the changes of *Ctr1*, *Ctr2*, *Ccs*, *Cox17*, and *Mt1a* genes’ expression during ETCM→ATCM transition were studied for the first time (Fig 3). In newborns, *Ctr1* gene activity in hepatocytes was very low. We think that at least two causes can be responsible for low activity of *Ctr1* gene. First, the expression of *Sp1* gene encoding a specific positive transcription regulator of the *Ctr1* gene activity is repressed during the ETCM, because intracellular copper concentration is high [73]. This assumption is supported by data that the Sp1 protein concentration in the liver nuclei is very low in the newborn, but not adult rats [74]. Second, the expression of *Ctr1* and *Atp7b* genes is related. This suggestion is based on the data that mice with selective *Atp7b* gene knockout in the liver (an animal model of WND) presented low *Ctr1* expression in hepatocytes as compared to wild littermates [70]. In any case, during ETCM, *Ctr1* gene activity is almost fully repressed. Possible, due to low activity of *Ctr1* gene, activities genes encoding Cu-chaperons, which transfer copper to apo-cuproenzymes (CCS to SOD1, COX17 to COX and ATOX/ATP7B to Cp), are low too, because these proteins closely interact during the transfer of copper. So, metallation of SOD1 required the CCS:SOD1 complex to be bound to the bilayer and CCS to interact with the CTR1 [75]. Hence, CCS-dependent copper acquisition needs *Ctr1* gene activity. Perhaps, during the ETCM, apo-SOD1 metallation is carried out by a CCS-independent pathway, and copper is delivered to SOD1 by the glutathione that acquires copper from MT [76]. For this assumption, it is important that MT expression is high during ETCM (Fig 3). Interestingly, that expression of SOD1 and COX4 was significantly increased during ETCM, but in the same time CCS and COX17 expression levels were remained low. It is agrees with the idea that MT is copper donor for metallation these cuproenzymes in newborns. In adult rats, CCS-mRNA level increased strongly. It means that, in ATCM, the apo-SOD1 started to get copper from the Cu(I)-CCS. It is possible, ATOX1 (copper provider for ATP7B) and COX17 (copper provider for COX) acquire copper in the ways similar to CCS [77, 78].

### (iv) A work hypothesis, why CTR1 does not play role of the main copper importer during ETCM

**Fig 4:**
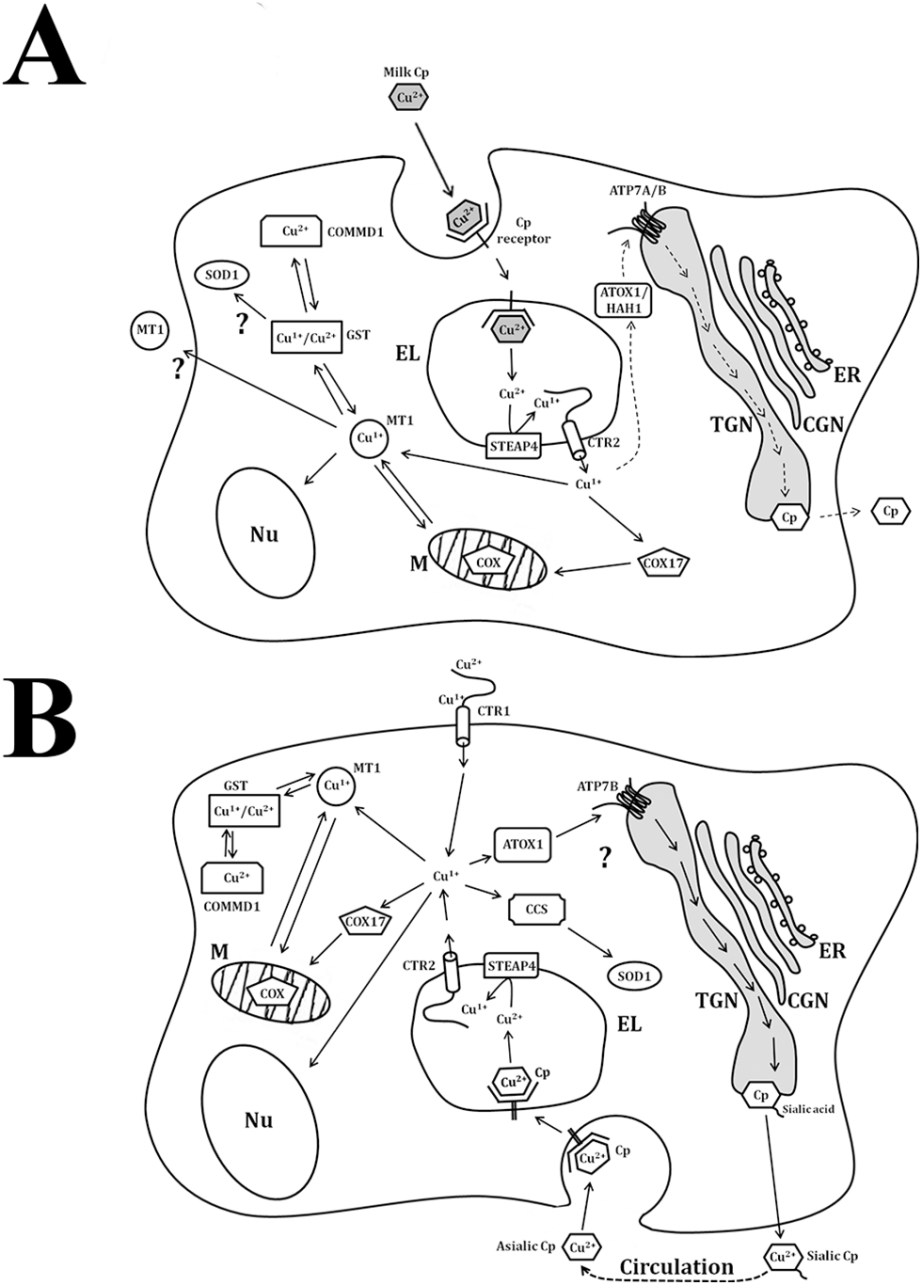
Schema illustrating copper turnover in the hepatocytes newborns (A) and adults rats (B). (A) In newborns, milk Cp enters to gastrointestinal tract and due to transcytosis transfers into bloodstream, and then it binds with hepatic Cp receptor and proceeds into endolysosomes (EL). At pH > 5, Cu(II) ions are dissociated from milk Cp molecule, Cu(II) is reduced to Cu(I) by STEAP4 and imported by CTR2 in cytosol. Here, Cu(I) is redistributed between Cu(I)-chaperons to be delivered to the places of apo-cuproenzymes formation (trans Golgi network (TGN), cytosol, mitochondria (M)). Also copper is bound with MT, and involved to redox cycle MT/glutathione or delivered to nucleus (Nu) and M as well as exported to extracellular space. As MT is found in mitochondria and nucleus [40], possibly, it transferred copper to the nucleus and mitochondria (or brings copper to their cytosolic surface). (B) In adults, absorbed nutrient copper is imported by CTR1 and distributed between Nu, M, cytosol, and MT [5]. Copper of disturbed cuproenzymes can be re-cyclized via endocytosis or autophagy. In both cases, the copper return in metabolic cycle through STEAP4/CTR2 endolysosomal system.

In newborns, the main source of nutrient copper is milk Cp, which is synthesized in mammary glands and secreted into milk [27, 79–81]. Milk Cp contains the major fraction of the milk copper [27], and it is transferred from the gastrointestinal tract to the bloodstream by transcytosis [27, 82]. Then the liver cells uptake milk Cp *via* endocytosis using a specific Cp receptor that is expressed in the hepatocytes of newborns, but not in adults [82, 83]. Accordingly, milk Cp is imported into the endosomes/lysosomes compartment. In lysosomes, at low pH, copper dissociates from the Cp and is reduced to Cu(I) by the Fe/Cu-reductase STEAP4 [84]. After Cu(I) is bound by the copper-binding motifs of CTR2, which takes part in the lysosomal copper turnover [85], and transfers copper to cytosol. There copper is distributed according to the cellular requirements. A model of the putative pathway involving CTR2 in the copper turnover is presented in Fig 4A. It explains the disproportion in *Ctr1* and *Ctr2* genes activities in the liver of newborns (Fig 3A). In the adult liver, CTR2 continues to take part in the copper re-cyclization from the asialic Cp, which is taken up from the bloodstream through asialoglycoprotein receptors [86]. However, the main copper import pathway in adult rats is going through CTR1; at this time the expression level of the *Ctr1* gene increases strongly (Fig 4B).

In summary, the data obtained in this work reveal the new aspects of copper metabolism during ETCM. They allow us to suggest that at this time in the liver *(i)* mechanisms of copper accumulation and its redistribution in organelles parallels those in patients with Wilson disease; *(ii)* in newborns, CTR1 is neither a major importer of copper no a copper donor for Cu(I)-chaperons; *(iii)* gene expression profile of a copper transporting proteins is adapted to the main nutrition source of copper, *i*.*e*. milk Cp. The data on the relation between the copper nutrient source and the profile of gene expression related to copper-transport further emphasize the importance of maintaining a proper dietary copper balance in newborns and emphasizes the value of milk Cp as a food source of copper for newborns.

## Abbreviations

P: postnatal development
P3, P6, *etc*.: age in days
ETCM: embryonic type copper metabolism
ATCM: adult type copper metabolism
Cp: ceruloplasmin
COX: cytochrome-c-oxidase
COX17: COX copper chaperone
MT: metallothionein
SOD: superoxide dismutase
CCS: copper chaperon of superoxide dismutase
ATP7A(B): copper transporting P1 ATPases
COMMD1: copper metabolism (Murr1) domain-containing protein
CTR1(2): copper transporters
WB: Western Blot analysis
